# Understanding social inequalities in children being bullied: UK Millennium Cohort Study findings

**DOI:** 10.1371/journal.pone.0217162

**Published:** 2019-05-29

**Authors:** Melisa Campbell, Viviane S. Straatmann, Eric T. C. Lai, Joanne Potier, Snehal M. Pinto Pereira, Sophie L. Wickham, David C. Taylor-Robinson

**Affiliations:** 1 University of Liverpool, Public Health and Policy, Liverpool, United Kingdom; 2 Alder Hey Children’s NHS Foundation Trust, Liverpool Fresh Child and Adolescent Mental Health Services (CAMHS), Liverpool, England, United Kingdom; 3 University College London, Institute of Epidemiology and Health Care, London, United Kingdom; Leibniz Institute for Prevention Research and Epidemiology BIPS, GERMANY

## Abstract

**Background:**

Children living in disadvantaged socio-economic circumstances (SEC) are more commonly victims of bullying, but pathways leading to social inequalities in being bullied are unclear. We assess how early life risk factors might mediate the increased risk of being bullied at age seven for children living in disadvantaged circumstances.

**Material and methods:**

Using data from 5,857 children in the UK Millennium Cohort Study (MCS) we calculate risk ratios (RR) for being bullied at age seven (child-reported), by household income quintile. Socially patterned risk factors for being bullied relating to social networks, family relationships and child characteristics from birth to age five were adjusted for to assess if they mediated any association between SEC and being bullied.

**Results:**

48.6% of children reported having been bullied. Children living in the lowest income households were at 20% greater risk of being bullied compared to those from the highest (RR1.20, 95%CI 1.06,1.36). Controlling for social networks, family relationships and child characteristics attenuated the increased risk for children in low income households to aRR 1.19 (95%CI 1.05, 1.35), aRR 1.16 (95%CI 1.02,1.32) and aRR 1.13 (95%CI 1.00,1.28) respectively. Our final model adjusted for risk factors across all domains attenuated the RR by 45% (aRR 1.11,95%CI 0.97,1.26).

**Conclusions:**

About half of children reported being bullied by age seven with a clear social gradient. The excess risk in children growing up in disadvantaged circumstances was partially explained by differences in their early years relating to their social network, family relationships and the child’s own abilities and behaviours. Policies to reduce inequalities in these risk factors may also reduce inequalities in the risk of being bullied in childhood.

## Introduction

Bullying is a common international public health problem, more frequently experienced by children growing up in disadvantaged circumstances.[1,2] Being bullied is characterised by being subject to negative overt and/or covert behaviours, involving a real or perceived power imbalance, delivered physically (e.g. hitting), emotionally (e.g. stigmatising) and/or socially (e.g. excluding). [3,4]

Children who experience being bullied at primary school age are more likely to be bullied as a teenager and both increase the risk of victimization as an adult.[5] Those subjected to bullying at any age are more likely to experience a range of poorer health and social outcomes subsequently[1,2,4,6] including lower self-esteem,[7] poorer mental health,[8] poorer physical health,[9] poorer quality adult relationships[7] and lower educational attainment.[10] In addition, they are also more likely to exhibit anti-social behaviours in later life including committing crimes[9,10] and are at increased risk of attempting and completing suicide. [4,11,12]

Tippet et al [1] in their recent meta-analysis found a 40% higher odds of being bullied in children from the lowest income households, compared to the wealthiest. Their study highlights the need to better understand the early life origins for pathways driving social inequalities in being bullied in childhood.[1]

The pathways through which socio-economic circumstances (SECs) influence health and wellbeing are complex, but ultimately result in unfair and unjust difference in power and resources available to economic, material and psychosocial conditions.[13] Children born into poverty are significantly disadvantaged throughout their entire life-course experiencing worse physical and mental states of health and wellbeing including bully victimization.[2] To tackle social inequalities, it is necessary first to understand their pathway and potential mediators in order to ameliorate risks.[13]

The social-ecological systems perspective[14] provides a framework for our analysis. It suggests that social behaviour patterns, including being bullied, are shaped by social and environmental conditions that evolve across the life-course.[14,15] In contemporary literature, socially patterned risk factors for being bullied relate to: the child's social networks[16] and their family[17,18]. However, there is a focus on the child themselves in order to explain differences in bullying victimization.[19]

It is unclear the extent to which these factors explain the increased risk of being bullied for children living in more disadvantaged SECs. Using a contemporary, nationally representative sample of children from the UK, we therefore aimed to assess the social patterning of being bullied by age seven years old. We also examined the extent to which any excess risk in being bullied for children growing up in disadvantaged circumstances was explained by factors in their early life social networks, the child’s family relationships, and the child’s characteristics and abilities.

## Materials and methods

### Design, setting, and data source

We used data from the UK Millennium Cohort Study (MCS), a nationally representative sample of 19,250 children born in the UK between September 2000 and January 2002, sourced from UK Data Service in 2015.[20] There were 13,681 interviews at age seven years and of these, 12,222 (89%) singleton born children had responses to questions we have used as our primary outcome (i.e. experience of being bullied) and exposure (i.e. household income reported by parent), meeting our inclusion criteria. To control for potential differences in the relationship between our identified mediating risk factors and multiple births, [21] we only use singleton births. In our complete case analysis we had a sample of 5,857 children with data on all variables of interest. The MCS used a stratified clustered sampling design applied to child’s residential electoral wards; this provided rural and urban representation.[22] The study oversampled children living in disadvantaged areas and, in the case of England, areas with high proportions of ethnic minority groups by means of its stratified clustered sampling design.[22] Information on the cohort and sampling design can be found in the cohort profile[22] or online (www.cls.ioe.ac.uk/mcs). The MCS was approved by the South West and London Multi-Centre Research Ethics Committees (MREC/01/6/19, MREC/03/2/022, 05/MRE02/46 for sweeps one, two and three respectively).[23] The Millennium Cohort Study obtained informed written consent from parent/ guardians of the cohort children in order to participate in the study, children themselves as they grow-up and other participants as necessary.[23] For the present study the MCS has been fully anonymised and did not require additional ethics approval or consent for further analysis.

### Primary outcome and exposure

The primary outcome was a measure of whether the MCS child had ever been bullied, derived from the child’s own response to the question “How often do children bully you in school?” asked at age seven. We regrouped responses into a binary variable for our primary analysis (ever = 'all of the time' and 'some of the time' or never bullied = 'never'). The question was asked by a trained interviewer as part of a longer interview with the MCS child using a questionnaire and this was usually conducted in their home. No formal definition of bullying was provided to the child.

Our primary SEC exposure was equivalised household income (EHI) measured by parents reporting their household income, recorded when children were nine months old, weighted for number of adults and dependent children in the household, and divided into quintiles. This provides a stable measure of early life SEC that preceded the mediator and outcome measures,[24] providing temporal sequencing to assess mediating pathways over the life-course.

### Early life risk factors for being bullied in childhood

We identified early life risk factors for being bullied in childhood that are available in MCS. Such factors include the connectedness of the child’s social network, including their social support[25] and social activities like clubs[26]; family relationships which will shape the child's self-esteem and social identity including family break-up including those resulting from household abuse, negative relationships[27,28] or authoritarian parenting;[17] and the child's abilities[29] and behaviours[16] that impact on social skills and resilience.

#### Factors that may influence the child’s early life social network

Factors under this category included: child-reported having at least one close friendship at age five years old (no, yes);[30] child-report of play with children outside of school at age five years old (daily, weekly, monthly = yes or never = no);[30] main responder-reported child participation in sport activities at age five years old (3 or more days/week, 2 days/week, 1 day/week, less often, none);[16,26] main responder-reported moved school at age five years old; and main responder-reported social network surrounding family at age five years old (both family and friends nearby, family only, friends only, neither).[25]

#### Factors that may influence the child’s earlier life family relationships

Factors under this category included: siblings/other children in the house when the MCS child was born (no: only child, 2 or 3+ other children);[28] quality of main responder (usually the mother) and also their partner (usually the father or father figure) relationship with MCS child both measured using the Pianta Short Form[31] (warm/mostly warm, distant) at three years old; parenting style at age three years old (Formal/ rules, Informal/no rules); family break up from age three and five years (yes, no);[16] main responder (usually the mother) and also their partners their partner (usually the father or father figure) distress level, both measured using the Kessler score for mental distress in the last month, assessed at MCS child's age three years (score≤5: normal; score>5: distressed); indoor activities with family as a measure of spending positive times with family at age five years old (daily/several times per week, a couple of times per week, monthly, annually/never); and main responders use of smacking at five years old (never, less than monthly, more than monthly/ daily).[17]

#### Child’s earlier life abilities and behaviours

Factors under this category included: socio-emotional development using the total score from the Strength and Difficulties Questionnaire (SDQ) ((normal (0 to 13), borderline (14 to 16), and abnormal (17+)). The SDQ assesses socio-emotional behavioural problems through five domains relating to peer problems, conduct disorders, hyperactivity, emotional problems, and pro-social behaviours measured at five years based on activities within the last 6 months—we used the total SDQ score, which excludes prosocial.[16] Bracken school readiness at school entry (school ready (>80 mean score), not school ready (0 to 79 mean score)) which measures cognitive ability at age three years old related to colour recognition, upper and lower case letters, numbers and counting, describing sizes in words, comparisons and naming basic shapes;[16] parent-reported child diagnosis of a limiting long term illness/condition at five years old (no, yes);[29] and finally, child overweight or obese at age five years old defined using the age and sex specific International Obesity Task Force (IOTF) cut-offs.[32]

#### Analysis strategy and statistical methods

Our analysis progressed in three stages. First, we produced descriptive statistics for the prevalence of being bullied at seven years old according to household income quintiles when the child was 9 months old, based on data meeting our inclusion criteria. Secondly, we undertook a univariable analysis to explore and assess the strength of association between household income (exposure) and child-reported being bullied (outcome) with all the early life risk factors as listed above. We did this by estimating relative risk ratios (RR) and 95% confidence intervals (95%CI) by Poisson regression. We considered factors as potential mediators for our third analysis stage, based on their significance level in the univariable analysis, calculated using the likelihood ratio test (*p =* ≤0.1). Thirdly, we conducted a complete case multivariable analysis adding our potential mediators grouped into the three domains most commonly reported: factors influencing the child’s social networks, the child’s family relationships and also the individual child’s abilities and behaviours. We used the World Health Organisation’s ‘stepwise approach’ for tackling social inequalities which seeks to reduce the gap between most advantaged and most disadvantaged.[33] Applied to our analysis, we assessed the change to the SEC gap, by comparing the risk for children in the lowest income quintile with the highest, from a sex and ethnicity adjusted baseline. We calculated change in the adjusted baseline RR comparing children in the lowest income quintile with the highest calculated ((adjusted baseline RR)- adjusted RR)/(adjusted baseline RR-1)*100).[34] We visualized the change in RRs comparing children living in the lowest income quintile families to the highest (the SEC gap). All analyses were conducted in Stata/SE v.13 (Stata Corporation, College Station, TX, U.S.A.) with survey (svy) commands to account for the sample design and attrition up to age seven. We estimated all model parameters by maximum likelihood and used Wald tests to assess the significance of individual model parameters.

#### Robustness tests

We repeated our analysis using multiple imputation by chained equations (MICE) to assess the impact of missing data on risk factors in our complete case analysis; missing data ranged from zero for MCS child’s sex to 37.6% for the Pianta scale at 3 years (n = 4597). (S1 Fig). Twenty imputed datasets were calculated using 12 variables in the final multivariable regression model including our study outcome (being bullied at seven years old), primary exposure (household income quintile) and child’s sex. MICE estimates were combined using Rubin’s rules.[35]

We also repeated the analysis with an alternative measure of SECs based on highest maternal educational qualification at birth of cohort child, comparing those with degree plus to those with no formal qualification (none).

We repeated our analysis using an alternative outcome of being persistently bullied at seven years old, comprising children who reported being bullied daily or weekly. Those who were less frequently or never bullied created the comparison group (not persistent = some of the time and never). We also repeated our analysis without our partner reported measure, to account for those children living without a father or father figure to include those living in lone parent households.

We undertook an additional counterfactual mediation analysis to assess how much of the effect of SEC income on being bullied in childhood is mediated via our domains of risk factors. Unlike previous mediation methods, this newer approach can accommodate multiple and correlated mediators. [36] Using the complete case sample we estimated the Natural Direct Effect (NDE), Natural Indirect Effect (NIE) and Total Effect (TE), after adjusting for sex and ethnicity at baseline, using the *Medflex* package (2018) in R.[36]

## Results

Overall, by seven years of age, 48.6% (n = 5,973) of children self-reported being bullied. There was a social gradient; 52.9% in the lowest income quintile reported having been bullied, compared to 43.9% in the highest (Table 1). All the potential mediators for being bullied demonstrated significant social patterning and were progressed onto the univariate analysis, with the exception of paternal warmth (p = 1.74), (Table 1).

**Table 1:** Characteristics of the study population meeting inclusion criteria, by household income quintile at birth of child.

Variable [p-value]	Income quintiles % (n)					
	1 (Lowest income)	2	3	4	5 (Highest income)	OVERALL
Number of children	2567	2574	2419	2399	2263	12,222
**OUTCOME VARIABLES**						
*MCS child reported been bullied by age 7 (primary outcome)* [**p = <0.001]**						
Never	47.1 (1208)	50.5 (1300)	50.7 (1226)	53.5 (1283)	56 (1268)	51.4 (6285)
Ever	52.9 (1359)	49.5 (1274)	49.3 (1193)	46.5 (1116)	43.9 (995)	48.6 (5937)
*MCS child reported persistently being bullied by 7yrs old (alternative outcome)* [**p = <0.001]**						
Never or infrequent	86.2 (2214)	89.4 (2302)	91.6 (2215)	93.3 (2238)	95 (2150)	91 (11119)
Daily/ weekly	13.8 (353)	10.6 (272)	8.4 (204)	6.7 (161)	5 (113)	9 (1103)
**PRIOR FACTORS—***to be adjusted for at baseline*						
*Child's sex (parent reported)* [**p = 0.4863]**						
Male	49.1 (1260)	49.8 (1282)	51.8 (1252)	49.6 (1190)	50.7 (1147)	50.2 (6131)
*Child*: *minority/ ethnic at birth (parent reported)* [**p = <0.001]**						
Other	26.0 (565)	20.6 (476)	9.2 (206)	6.6 (149)	8.3 (179)	14.2 (1575)
**FACTORS INFLUENCING SOCIAL NETWORK**						
*Child friendships at 5yrs old (parent reported)* [**p = <0.001]**						
Has no close friends	2.9 (64)	2.5 (57)	1.7 (38)	1.4 (32)	1.0 (21)	1.9 (212)
*Like to play with friends outside of school at 5yrs old (parent reported)* [**p = <0.001]**						
No	18.6 (438)	16.3 (384)	9.4 (229)	6.7 (160)	3.9 (91)	10.1 (1551)
*After school sport at 5yrs old (parent reported)* [**p = <0.001]**						
3 or more days per week	71.5 (1667)	63.5 (1507)	44.1 (1006)	30.8 (742)	22.6 (508)	46.6 (5430)
2 days per week	3.7 (89)	5.1 (138)	8.9 (218)	13.6 (330)	17.1 (392)	9.6 (1167)
1 day per week	6.9 (169)	7.8 (215)	16.1 (379)	21.4 (489)	27.5 (595)	15.9 (1847)
Less often	17.9 (440)	23.6 (575)	30.9 (722)	34.2 (775)	32.7 (712)	27.9 (3224)
*Family's social network at 5yrs old (parent reported)* [**p = <0.001]**						
Both family and friends nearby	51.2 (1258)	53.2 (1335)	55.8 (1271)	50.9 (1190)	40.7 (885)	50.4 (5939)
Family nearby	12.6 (287)	9.6 (249)	9.0 (226)	6.8 (182)	5.2 (136)	8.6 (1080)
Friends nearby	20.0 (471)	23.4 (548)	24.2 (561)	30.1 (677)	39.6 (862)	27.4 (3119)
No family and friends nearby	16.3 (345)	13.8 (301)	11.6 (266)	12.2 (287)	14.5 (323)	13.6 (1522)
*Moved school ever at 5yrs old (parent reported)* [**p = 0.0021]**						
Yes	4.4 (82)	3.2 (60)	2.8 (54)	2.1 (41)	2.1 (40)	2.9 (277)
**FACTORS INFLUENCING EARLIER LIFE FAMILY RELATIONSHIPS**						
*Number of children in household at MCS birth (parent reported)* [**p = <0.001]**						
Only MCS child	38.9 (998)	31.4 (809)	40.2 (973)	44.4 (1066)	56.4 (1276)	41.9 (5122)
2 children in household (inc. MCS child)	47.0 (1207)	56.2 (1446)	54.4 (1317)	52.9 (1270)	41.8 (946)	50.6 (6186)
3+ children in household (inc. MCS child)	14.1 (362)	12.4 (319)	5.3 (129)	2.6 (63)	1.8 (41)	7.5 (914)
*Main responder's relationship at 3yrs old (parent response to Pianto scale)* [**p = <0.001]**						
Not warm	2.1 (38)	1.2 (26)	0.9 (18)	0.4 (14)	0.3 (11)	1.0 (107)
*Paternal relationship at 3yrs old (parent response to Pianto scale)* [**p = 0.0839]**						
Not warm	1.7 (15)	1.4 (18)	1.7 (21)	1.1 (19)	0.6 (13)	1.2 (86)
*Parenting style at 3yrs old (parent reported)* [**p = <0.001]**	* *	* *	* *	* *	* *	* *
Formal	33.4 (692)	37.1 (836)	42.6 (919)	50.4 (1087)	55.1 (1128)	43.9 (4662)
Informal	66.6 (1354)	62.9 (1356)	57.4 (1257)	49.6 (1125)	44.9 (985)	56.1 (6077)
*Family break-up from 3 or 5yrs old (parent reported)* [**p = <0.001]**						
Yes	13.9 (327)	15.4 (336)	12.5 (268)	7.9 (175)	7.3 (145)	11.5 (1251)
*Main responder's levels of distress at 5yrs old (parent response to Kessler scale)* [**p = <0.001]**						
Distressed (Kessler score >5)	29.4 (625)	25.1 (551)	16.9 (361)	13.1 (291)	9.6 (206)	18.6 (2034)
*Partner levels of distress at 5yrs old (parent response to Kessler scale)* [**p = <0.001]**						
Distressed (Kessler score >5)	23.0 (235)	22.0 (318)	15.2 (268)	12.3 (240)	11.4 (215)	15.7 (1276)
*Smacking used as discipline at 5yrs old (parent reported)* [**p = <0.001]**	* *	* *	* *	* *	* *	* *
Never	46.1 (978)	44.5 (997)	40.5 (913)	41.3 (948)	48.5 (1053)	44.0 (4889)
Less than monthly	52.2 (1090)	53.4 (1206)	58.3 (1313)	57.5 (1322)	51.2 (1118)	54.5 (6049)
More than monthly/ daily	1.7 (35)	2.1 (47)	1.2 (37)	1.2 (32)	2.5 (14)	1.3 (165)
*Frequency family indoor activities at 5yrs old (parent reported)* [**p = <0.001]**						
Daily/several times per week	75.7 (1786)	72.25 (1747)	73.9 (1720)	74.5 (1744)	74.4 (1655)	74.2 (8652)
Monthly/every few months	22.1 (536)	25.6 (639)	24.7 (579)	24.9 (577)	25.3 (545)	24.5 (2876)
Annually/ never	2.2 (43)	2.2 (48)	1.4 (26)	0.5 (15)	0.3 (6)	1.3 (138)
**FACTORS FOR EARLIER LIFE ABILITIES AND BEHAVIOURS**						
*Child is school ready at 3yrs old (Bracken measure applied)* [**p = <0.001]**						
No	25.9 (497)	18.0 (390)	9.2 (181)	5.2 (116)	2.9 (63)	12.0 (1247)
*Strength and Difficulty Questionnaire (SDQ) score at 5yrs old* [**p = <0.001]**						
Average	79.8 (1753)	84.2 (1991)	91.9 (2116)	93.9 (2181)	96.2 (2107)	89.3 (10148)
Borderline	8.7 (205)	8.0 (174)	4.6 (107)	3.7 (83)	2.7 (59)	5.5 (628)
Below average	11.4 (243)	7.8 (162)	3.5 (66)	2.4 (53)	1.2 (31)	5.2 (555)
*Child has a limiting long standing illness at 5yrs old (parent reported)* [**p = <0.001]**						
Yes	22.3 (527)	18.5 (473)	19.7 (443)	16.4 (385)	16.5 (358)	18.7 (2186)
*Child's BMI status at 5yrs old (International Obesity Task Force thresholds applied)* [**p = <0.001]**						
Normal	54.9 (973)	49.7 (989)	55.0 (1074)	61.6 (1239)	68.3 (1341)	58.1 (5616)
Overweight	26.1 (503)	29.6 (594)	28.0 (575)	24.9 (507)	22.2 (454)	26.1 (2633)
Obese	18.9 (356)	21.0 (421)	17.1 (337)	13.5 (289)	9.5 (186)	15.8 (1589)

For complete case, see S1 Table in the supplementary documents.

In our univariate analysis household income and being bullied was significantly associated with all potential mediators at p-value less than 0.1, with the exception of four variables (For full univariate results see S2 Table). Moving school at five years old (p = 0.56), parenting style at three years old (p = 0.63), maternal warmth at five years old (p = 0.16) and child has a limiting longstanding illness at five years old (p = 0.13) were not independently associated with household income as a measure of SEC.

### Association between SECs and being bullied, adjusting for potential mediators

Our adjusted baseline model shows 20% greater risk of being bullied in the lowest household income quintile compared to the highest, (sex/ethnicity adjusted baseline RR 1.20, 95%CI 1.06,1.36) using the complete case sample.(Table 2) We note children from all other income groups had a higher risk of being bullied compared to those from the most affluent homes (quintile 5).

**Table 2:** Risk ratios (RR) 95% confidence intervals final multivariable analysis based on complete case analysis.

Bullying at age 7 years old	Adjusted baseline* [p = 0.003]			MODEL 1* [p = 0.008]			MODEL 2 * [p = 0.054]			MODEL 3* [p = 0.052]			FINAL MODEL*[p = 0.123]		
Household income quintile at birth	RR	95% LCI	95% UCI	RR	95% LCI	95% UCI	RR	95% LCI	95% UCI	RR	95% LCI	95% UCI	RR	95% LCI	95% UCI
Highest (Reference)	Ref	-	-	Ref	-	-	Ref	-	-	Ref	-	-	Ref	-	-
4	1.05	0.96	1.15	1.05	0.96	1.15	1.04	0.95	1.14	1.04	0.95	1.14	1.04	0.95	1.14
3	1.16	1.05	1.27	1.16	1.05	1.28	1.15	1.04	1.26	1.14	1.03	1.25	1.14	1.03	1.26
2	1.17	1.06	1.30	1.16	1.05	1.28	1.14	1.03	1.26	1.13	1.02	1.25	1.11	1.00	1.23
Lowest	1.20	1.06	1.36	1.19	1.04	1.35	1.16	1.02	1.32	1.13	1.00	1.28	1.11	0.97	1.27
**FACTORS INFLUENCING EARLY LIFE SOCIAL NETWORK**															
Child friendships at 5yrs old (parent reported) (Ref: yes)				1.20	0.90	1.59							1.28	0.97	1.69
Like to play with friends outside of school at 5yrs old (Ref: yes)				0.96	0.85	1.09							0.98	0.86	1.10
After school sport at 5yrs old (Ref: 3 or more days per week)				Ref	-	-							Ref	-	-
2 days per week				0.97	0.86	1.10							0.99	0.88	1.11
1 day per week				0.90	0.82	0.99							0.92	0.83	1.01
Less often				0.95	0.88	1.03							0.96	0.89	1.04
Family's social network at 5yrs old (Ref: family & friends nearby)				Ref	-	-							Ref	-	-
Family nearby				1.04	0.92	1.18							1.03	0.92	1.17
Friends nearby				1.09	1.01	1.18							1.10	1.02	1.18
No family and friends nearby				1.03	0.93	1.14							1.02	0.92	1.14
**FACTORS INFLUENCING EARLY LIFE FAMILY RELATIONSHIPS**															
Number of children at home at MCS birth (REF:MCS Child only)							Ref	-	-				Ref	-	-
2 children in household (inc. MCS child)							0.98	0.91	1.04				0.98	0.92	1.04
3+ children in household (inc. MCS child)							0.99	0.85	1.16				0.98	0.83	1.14
Main responders Kessler scale distress score at 5yrs old (Ref: ≤5 score)							1.09	1.00	1.19				1.05	0.96	1.15
Partner Kessler scale distress score at 5yrs old (Ref: ≤5 score)							1.06	0.97	1.16				1.05	0.97	1.15
Family break-up MCS child age 3 or 5yrs old (Ref: No)							1.46	1.22	1.75				1.41	1.17	1.69
Family indoor activities at 5yrs old (REF: Daily/several times p/w)							Ref	-	-				Ref	-	-
Monthly/every few months							1.05	0.98	1.12				1.05	0.98	1.12
Annually/ never							1.00	0.70	1.43				1.00	0.70	1.43
Smacking used as discipline at 5yrs old (REF: Never)							Ref	-	-				Ref	-	-
Less than monthly							1.08	1.01	1.15				1.07	1.00	1.14
More than monthly/ daily							1.20	0.94	1.54				1.15	0.89	1.49
**FACTORS FOR EARLY LIFE ABILITIES AND BEHAVIOURS **																
Child is school ready at 3yrs old (Ref: School ready)										1.05	0.95	1.16	1.04	0.94	1.15
Strength and Difficulty Questionnaire score at 5yrs old (REF: Average)										Ref	-	-	Ref	-	-
Borderline										1.33	1.17	1.51	1.28	1.13	1.46
Below average										1.20	1.02	1.40	1.16	0.99	1.36
Child's BMI status at 5yrs old (REF: Normal)										Ref	-	-	Ref	-	-
Overweight										1.08	1.00	1.17	1.08	1.00	1.16
Obese										1.06	0.97	1.16	1.06	0.97	1.15
**TOTAL COUNT**	** **	**5857**	** **	** **	**5857**	** **	** **	**5857**	** **	** **	**5857**	** **	** **	**5857**	** **

}*Adjusted (Adj.) baseline; MODEL 1 Adj. baseline & social network; MODEL 2 Adj. baseline & family relationships; MODEL 3 Adj. baseline & child's abilities and behaviours; Final Model Adj. baseline, social network.

**Fig 1:**
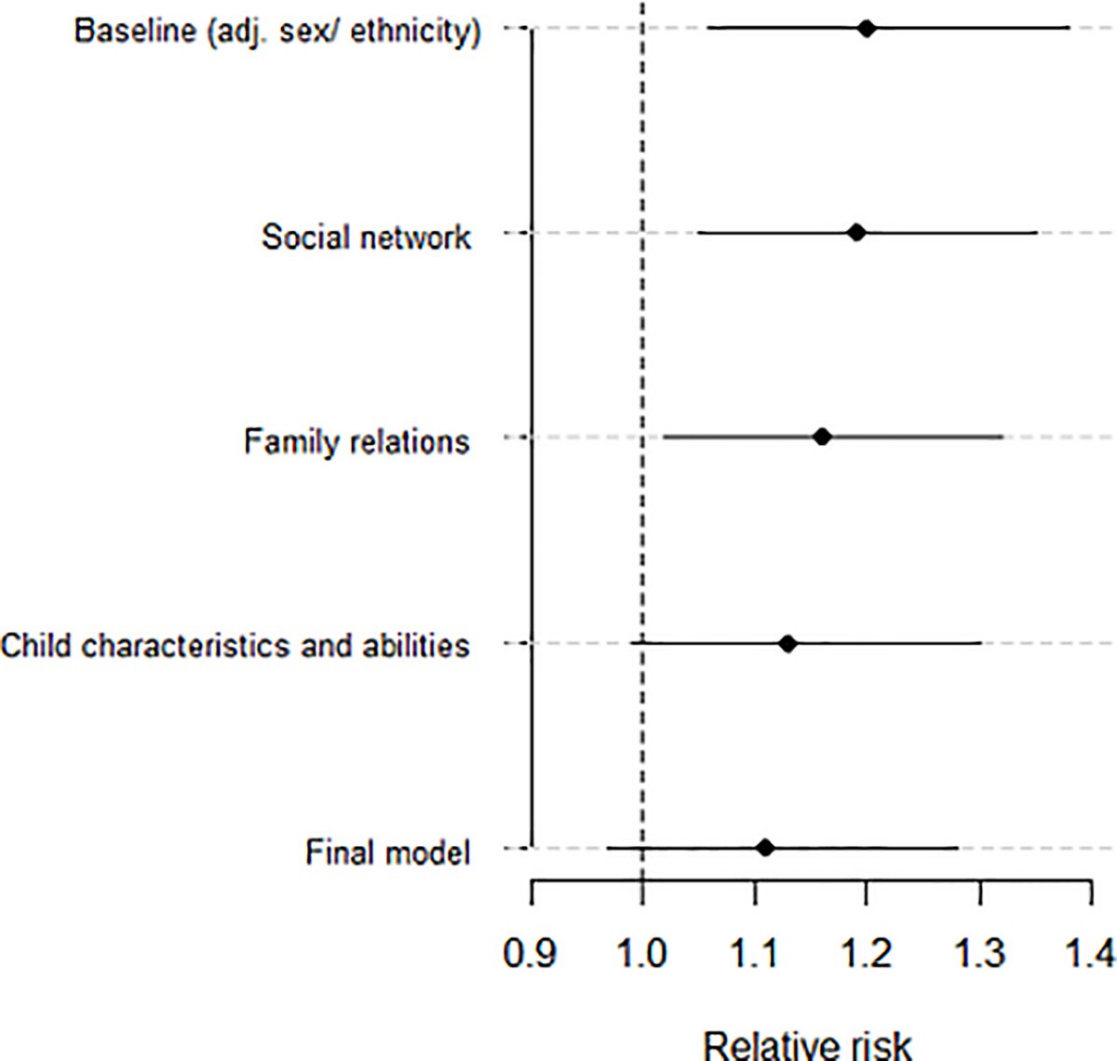
Relative risk changes for being bullied comparing lowest to highest income households in adjusted separate models.

Our first model adjusts for factors that may influence the child’s social networks attenuating the adjusted baseline increased risk in the most disadvantaged children by 5% (aRR 1.19, 95%CI 1.04, 1.35). Our second model adjusts for factors that may influence the child’s family relationships attenuating the adjusted baseline RR by 20% (aRR 1.16, 95%CI 1.02, 1.32). Our third model adjusts for the MCS child's abilities and behaviours attenuating the adjusted baseline RR by 35% (aRR 1.13, 95%CI 1.00, 1.28). (Fig 1). In the final model, adjusting for all factors potentially influencing the child's social network and family relationships, and their abilities and behaviours led to a 45% relative risk reduction compared to the baseline model, (aRR 1.11 95%CI 0.97 to 1.28, P = 0.123) rendering the association non-significant.(Fig 1)

In our multivariable model (Table 2), borderline SDQ score (aRR 1.28, 95%CI 1.13, 1.46), becoming a lone parent at MCS child age three to five years old (aRR 1.41, 95%CI 1.17, 1.69) and only having friends, rather than both family and friends living nearby (aRR 1.10, 95%CI 1.02, 1.18) remained statistically significantly associated with being bullied at age seven. We also note, that our model did not significantly attenuate the risk of bullying for children in middle income homes (Quintiles 2 & 3).

### Robustness tests

Findings from the MICE analysis yielded similar results, with a similar attenuation of 51.9% in the final model from a baseline aRR of 1.23 (95%CI 1.13, 1.34) to aRR 1.15 (95%CI 1.04, 1.27) after adjusting for all mediators in our final model. (S3 Table)

Our alternative outcome of persistent bullying was reported by one in ten children overall, and was also significantly higher in the lowest household quintile (14.6%, n = 353) compared to the highest (4.7%,n = 113) (RR 3.07, 95%CI 2.11, 4.47). Adjusting for all risk factors attenuated the RR by 52.6% (aRR 1.97, 95%CI, 1.27, 3.05) but the relative risk remained significant. (S3 Table) Using maternal educational qualifications as an alternative measure of SECs we found similar results. (S4 Table) Removing a partner reported measure we found similar results.(S4 Table).

Our counterfactual mediation analysis was comparable to our primary analysis. Overall 42.0% of the total effect of income (lowest income quintile versus highest) on risk of being bullied was mediated via early life factors related to the child’s social network, family relationships and abilities and behaviours.(S4 Table) This provides a natural direct effect of RR 1.16 (95%CI 1.05, 1.28) and natural indirect effect of RR 1.10 (95%CI 1.06, 1.14).

## Discussion

Using a nationally representative sample of UK children born in 2000, we found that 48.6% of children reported they had experienced being bullied by age seven years. The risk of ever being bullied by age seven was about 20% higher in the lowest compared to highest income quintile. Furthermore, the risk of being bullied daily or weekly was more three times greater. Adjusting for the child’s early life social network, family relationships and child abilities and behaviours attenuated this increased risk by about a half suggesting that addressing inequalities in these factors may reduce inequalities in being bullied in later childhood.

### Comparison with other findings

In our study using a representative UK sample of seven year olds around half reported experiencing some form of bullying. This broadly agrees with a UK government survey[37] that reports half of all 8–16 year olds have concerns about being bullied in school with nearly one in five having been regularly bullied in the last month.[37]

Research on early life drivers of social inequalities on later childhood risk of being bullied is scarce.[1,16] Numerous studies have identified low socio-economic status as a risk factor for being bullied.[1,28,38] A recent meta-analysis[1] of 28 international studies identified a 40% greater odds of being bullied for children growing up in poverty.[1] This was larger than the effect seen in our study—a 20% increase across income quintiles. Differences in the inequalities gradient in being bullied may reflect the outcome used, and the measure of SECs. In our study the social gradient was steeper when we used frequent bullying as the outcome.

Our study corroborates a smaller population-based cohort from the Environmental Risk (E-Risk) Longitudinal Twin study[28] of over 2000 children from the UK exploring the association between reports of being bullied and our three domains of proximal risk factors relating to the child’s life: school, neighbourhood, and family.[28] Unlike our study, the authors used parent and teacher reported prevalence of being bullied from 5 to 7 years old, which was much lower at 21.6%.[28] The E-Risk study provided a definition of bullying victimization to mothers, which may generate a more specific estimate compared to the question used in the MCS.(34,35). When a consistent definition of bullying is applied existing evidence suggests that maternal report of bullying replicates that of the child. [39]

Bowes et al found that school, neighbourhood, and family factors are independently associated with any involvement in bullying (including being victim to), above and beyond socio-demographic factors.[28] Similarly, we found a residual impact of SECs on the risk of being bullied after adjusting for the child's social network, family relationships and their abilities and behaviours. A smaller Dutch study[16] involving ~1000 children, explored the association between preschool behaviours, family characteristics (socio-economic status and family breakup) and parental mental health on bullying and being bullied.[16] The outcome was measured using peer nomination to identify both bullies and victims in the class, at ages 11 and 13.5 years old.[16] The researchers[16] reported preschool behavioural problems such as poorer motor skills and family relations including family break-up are important risk factors for being bullied in later childhood. We also found children with borderline socio-emotional behavioural difficulties, were more likely to be bullied, as were those who experienced a family break-up from 3 to 5 years old, independent of the child’s socioeconomic status.

### Strengths and limitations

We used a large nationally representative UK cohort and our results are likely to be generalisable to children today in the UK. Every two years from birth the MCS has consistently gathered extensive details on the MCS children, their family, and home and community environments using validated approaches, which has enabled us to explore a wide variety of covariates associated with the risk of being bullied.

A limitation is that the main outcome measure (e.g. having been bullied) is less ease to compare to other studies as it was reported by the child rather than an adult (e.g. a parent) close to them and no universal definition was applied. Although this approach has been widely used in other studies also exploring social inequalities [39] it may be that more children feel bullied than would be considered bullied by an adult or their teacher or definitions vary according to the responder, which is also commonly seen in studies with multiple reporting sources.[39,40] However, a potential strength is that the measure captures children’s own lived experience, and our child-reported prevalence of bullying compares with findings from UK surveys.[37]

The MCS surveys from birth to seven years old do not provide details on types or form (e.g. online or in person) where bullying may be experienced, highlighting an area unexplored by this study. Data collection from the MCS cohort at 14 years old provides details on where bullying is experienced including, cyber-risks. We recommend future research further explores the cyber-risks in explaining the social inequalities pathways leading to being bullied.

A limitation of our outcome is the lack of differential in frequency of being bullied. We captured this in our alternative outcome, and there was a much steeper social gradient in the experience of being bulled every day or weekly. Our study potentially underestimates baseline risk in being bullied by household income. Furthermore adjusting for several apiori- potential mediators attenuated the RR in our primary analysis to non-significant; we are aware, there may be other possible explanation for this attenuation. We strengthen our primary analysis by undertaking various robustness tests, measures to verify our results and underpinning assumptions. We repeated our analysis using a counterfactual mediation approach, more severe outcome measure and an alternative SEC exposure, which gave similar findings.

A further limitation was missing data, an inherent challenge in large cohort studies. We are aware, the children in the cohort with missing data could experience different pathways to being bullied. Reassuringly, using a multiple imputed dataset also produced similar results and conclusions. Furthermore, our analysis design may have excluded vulnerable groups (e.g. lone parent homes). We recommend future research should build upon our work, further explore pathways to social inequalities in children being bullied, to enable researchers and practitioners to understand them better.

Whilst the MCS provides a large dataset with extensive details on the child, their family and home, and community environments using validated approaches from birth, our research has been limited to exploring the mediating effects of variables available within. It is possible the remaining excess risk can be explained by variables not available within this dataset and future research should explore this further.

### Policy and practice implications

Tippet et al [1] highlighted that childhood SECs alone provide little policy guidance for targeting anti-bullying interventions amongst school age children.[1] By decomposing the pathways from disadvantaged SECs to increased risk of being bullied at early primary school age, we show that known pre-school risk factors can partially explain the increased risk in disadvantaged children. Many of these early life risk factors for being bullied are socially patterned, and are either preventable or their impact may be ameliorated with the right kind of support. Most current national anti-bullying interventions commence in primary schools, by which time socially patterned differences in the risk of being bullied have already been established. Our results thus suggest that efforts to tackle social inequalities in being bullied need to commence earlier, with an increased focus on supporting social network, family relationships and the child’s development from birth and throughout the pre-school years. Much of the excess risk of childhood SEC on bullying could be explained by other factors not available in the MCS dataset. We recommend further research is needed to better understand pathways to social inequalities in childhood bullying.

## Supporting information

S1 Fig Sample flow diagram. S1 Fig shows and accounts for missing and excluded data.
(DOC)

S1 Table Characteristics of the complete case population, by household income quintile at birth of child N = 5857. (DOC)

S2 Table Univariate analysis: Social patterning of risk factors for being bullied as a child. (DOCX)

S3 Table Robustness test for being bullied at age 7 and socio-economic circumstances (SEC) in the multivariable analysis stage. S3 Table presents the risk ratios (RR) 95% confidence intervals for multiple imputations by chained equations, alternative outcome and alternative exposures.
(DOC)

S4 Table Results from the formal counterfactual medication analysis (Medflex). S4 Table show the findings from applying a formal counterfactual medication analysis, an approach that gives us the flexibility to assess the effect of specific causal pathways in order to quantify its contribution to the outcome of interest.
(DOC)
