# Supplementary material for: Understanding social inequalities in children being bullied: UK Millennium Cohort Study findings
Source: PLoS One. 2019 May 29;14(5):e0217162. doi: 10.1371/journal.pone.0217162 (PMC6541267; doi:10.1371/journal.pone.0217162)
Supplement: S1 Fig — shows and accounts for missing and excluded data. (DOC) [file pone.0217162.s001.doc]

**S1 Fig**

Flow diagram accounting for missing data

**FINAL MODEL**

**COMPLETE CASE SAMPLE SIZE N=5,857**

**COMPLETE CASE**

**MISSING data: n=**6,365

*PRIORI: sex (n=489), minority/ ethnic (n=1,099),*

*SOCIAL****:*** *Has a close friendship at 5yrs (n=911), plays with friends outside of school at 5yrs (n=556*), *sports at 5yrs (n=554), indoor family activities at 5yrs (n=556), social network at 5yrs (n=562), school move at 5yrs (n=685).*

*FAMILY****:*** *number of children in household at birth (n=489), levels of maternal distress at 5yrs (n=1,068), Becomes a lone parent family 3-5years old (n=0) levels of paternal distress at 5yrs (n=4,068), child smacked at 5yrs (n=1,119), parenting style at 5yrs (n=1,483), maternal warmth at 5yrs (n=2,548), paternal warmth at 5yrs (n=4,597).*

*CHILD****:*** *School readiness at 3yrs (n=2,084), SDQ at 5yrs (n=891), BMI (n=2,771), LLTC at 5yrs (n=561), BMI at 5yrs (n=2,384).*

**SAMPLE SIZE: 5,857 *(47.9% OF THOSE MEETING THE INCLUSION CRITERIA)***

**INCLUSION CRITERIA**

**Primary outcome and exposure:** *ever/never bullied (n=975) household income (n=525)*

*CRITERIA: MCS primary socio-economic exposure question answered and child reported ever/never bullied in sweep 4.*

**SAMPLE SIZE: 12,222 *(89.3% OF THOSE RESPONDED SUCCESSFULLY ACROSS 4 SWEEPS)***

**SAMPLE ELIGIBLE FOR INCLUSION MCS SWEEP 4**

at age 7 years

(n= 17,031 households, response rate 88.7%)

(n=13,681 responded across all sweeps to 7 years)
