# Supplementary material for: Understanding social inequalities in children being bullied: UK Millennium Cohort Study findings
Source: PLoS One. 2019 May 29;14(5):e0217162. doi: 10.1371/journal.pone.0217162 (PMC6541267; doi:10.1371/journal.pone.0217162)
Supplement: S1 Table — (DOC) [file pone.0217162.s002.doc]

| S1 Table: Characteristics of the complete case population, by household income quintile at birth of child N=5857 | | | | | | |
| --- | --- | --- | --- | --- | --- | --- |
| **Variable [P-value]** | **Income quintiles % (n)** | | | | | **OVERALL** |
|  | **Highest 1** | **4** | **3** | **2** | **Lowest 5** |  |
|  |  |  |  |  |  |  |
| Number of children in complete case household quintile at birth | **1532** | **1484** | **1256** | **1056** | **529** | **5857** |
| **OUTCOME VARIABLES** | | | | | | |
| *MCS child reported been bullied by age 7 (primary outcome) [p=<0.001]* | | | | | | |
| Never | 57.6 | 55.6 | 51.1 | 50.3 | 49.3 | 53.7 |
| Ever | 42.4 | 44.4 | 48.9 | 49.7 | 50.7 | 46.3 |
| *MCS child reported persistently being bullied by 7yrs old (alternative outcome) [p=<0.001]* | | | | | | |
| Never or infrequent | 96.0 | 94.6 | 92.2 | 88.7 | 87.9 | 92.9 |
| Daily/ weekly | 4.0 | 5.4 | 7.8 | 11.3 | 12.1 | 7.1 |
| **PRIOR FACTORS** *- to be adjusted for at baseline* | | | | | | |
| *Child's sex (parent reported)** [p=0.7973]* | | | | | | |
| Male | 49.0 | 50.1 | 51.4 | 49.3 | 51.6 | 50.1 |
| *Child: minority/ ethnic at birth (parent reported) [p=<0.001]* | | | | | | |
| Other | 6.7 | 4.3 | 5.8 | 10.0 | 11.7 | 6.9 |
| **FACTORS INFLUENCING SOCIAL NETWORK** | | | | | | |
| *Child friendships at 5yrs old (parent reported) [p=<0.0094]* | | | | | | |
| Has no close friends | 0.5 | 1.3 | 1.5 | 1.7 | 2.5 | 1.3 |
| *Like to play with friends outside of school at 5yrs old (parent reported) [p=<0.001]* | | | | | | |
| No | 2.7 | 5.9 | 7.5 | 12.8 | 14.9 | 7.3 |
| *After school sport at 5yrs old (parent reported) [p=<0.001]* | | | | | | |
| 3 or more days per week | 21.1 | 28.1 | 40.1 | 57.0 | 65.5 | 37.0 |
| 2 days per week | 18.0 | 15.1 | 9.0 | 6.4 | 4.6 | 12.2 |
| 1 day per week | 28.1 | 21.4 | 18.0 | 8.4 | 9.1 | 19.2 |
| Less often | 32.9 | 35.4 | 33.0 | 28.3 | 20.7 | 31.7 |
| *Family's social network at 5yrs old (parent reported) [p=<0.001]* | | | | | | |
| Both family and friends nearby | 39.2 | 52.4 | 59.2 | 58.1 | 57.8 | 51.7 |
| Family nearby | 4.8 | 6.0 | 8.2 | 7.4 | 10.1 | 6.7 |
| Friends nearby | 41.8 | 30.5 | 23.3 | 22.9 | 19.0 | 29.7 |
| No family and friends nearby | 14.2 | 11.1 | 9.3 | 11.6 | 13.2 | 11.8 |
| *Moved school ever at 5yrs old (parent reported) [p=0.9558]* | | | | | | |
| Yes | 2.0 | 1.8 | 1.9 | 2.1 | 2.5 | 2.0 |
| **FACTORS INFLUENCING EARLIER LIFE FAMILY RELATIONSHIPS** | | | | | | |
| *Number of children in household at MCS birth (parent reported) [p=<0.001]* | | | | | | |
| Only MCS child | 54.0 | 41.2 | 38.2 | 27.0 | 38.3 | 41.3 |
| 2 children in household (inc. MCS child) | 44.4 | 56.4 | 55.6 | 61.9 | 50.4 | 53.4 |
| 3+ children in household (inc. MCS child) | 1.6 | 2.4 | 6.2 | 11.1 | 11.3 | 5.3 |
| *Family break-up from 3 or 5yrs old (parent reported) [p=<0.001]* | | | | | | |
| Yes | 0.3 | 0.7 | 1.1 | 3.5 | 3.1 | 1.4 |
| *Main responder's levels of distress at 5yrs old (parent response to Kesseler scale) [p=<0.001]* | | | | | | |
| Distressed (Kessler score >5) | 7.6 | 11.4 | 13.2 | 20.5 | 24.3 | 13.4 |
| *Partner levels of distress at 5yrs old (parent response to Kesseler scale) [p=<0.001]* | | | | | | |
| Distressed (Kessler score >5) | 11.5 | 12.4 | 14.4 | 21.8 | 23.5 | 15.2 |
| *Parenting style at 3yrs old (parent reported) [p=<0.001]* | | | | | | |
| Formal | 56.3 | 49.2 | 41.8 | 38.1 | 35.7 | 46.5 |
| Informal | 43.8 | 50.8 | 58.2 | 61.9 | 64.3 | 53.6 |
| *Smacking used as discipline at 5yrs old (parent reported) [p=0.0069]* | | | | | | |
| Never | 48.1 | 40.6 | 43.1 | 41.1 | 42.8 | 43.5 |
| Less than monthly | 51.3 | 58.5 | 55.7 | 57.5 | 56.0 | 55.5 |
| More than monthly/ daily | 0.6 | 0.9 | 1.3 | 1.5 | 1.2 | 1.0 |
| *Frequency family indoor activities at 5yrs old (parent reported) [p=0.0021]* | | | | | | |
| Daily/several times per week | 73.8 | 75.7 | 73.6 | 72.7 | 76.6 | 74.3 |
| Monthly/every few months | 25.9 | 23.9 | 25.4 | 25.5 | 21.4 | 24.8 |
| Annually/ never | 0.3 | 0.4 | 1.1 | 1.8 | 2.0 | 0.9 |
| **FACTORS FOR EARLIER LIFE ABILITIES AND BEHAVIOURS** | | | | | | |
| *Child is school ready at 3yrs old (Bracken measure applied) [p=<0.001]* | | | | | | |
| No | 2.7 | 4.8 | 7.8 | 14.2 | 24.6 | 8.2 |
| *Child's BMI status at 5yrs old (International Obesity Task Force thresholds applied) [p=<0.001]* | | | | | | |
| Normal | 67.9 | 62.7 | 54.4 | 50.2 | 51.6 | 59.2 |
| Overweight | 22.4 | 23.6 | 28.1 | 29.6 | 27.7 | 25.6 |
| Obese | 9.7 | 13.7 | 17.6 | 20.2 | 20.8 | 15.2 |
| *Child has a limiting long standing illness at 5yrs old (parent reported) [p=<0.001]* | | | | | | |
| Yes | 15.6 | 15.3 | 18.7 | 16.3 | 25.7 | 17.2 |
| *Strength and Difficulty Questionnaire (SDQ) score at 5yrs old [p=<0.001]* | | | | | | |
| Average | 97.1 | 95.3 | 94.1 | 88.8 | 80.9 | 93.1 |
| Borderline | 2.2 | 3.1 | 3.4 | 5.1 | 9.0 | 3.8 |
| Below average | 0.7 | 1.7 | 2.5 | 6.1 | 10.1 | 3.1 |
