# Supplementary material for: Understanding social inequalities in children being bullied: UK Millennium Cohort Study findings
Source: PLoS One. 2019 May 29;14(5):e0217162. doi: 10.1371/journal.pone.0217162 (PMC6541267; doi:10.1371/journal.pone.0217162)
Supplement: S2 Table — (DOCX) [file pone.0217162.s003.docx]

**S2 Table** Univariate analysis: social patterning of risk factors for being bullied as a child.

| **OUTCOME VARIABLES** | **RR** | **L95% CI** | **U95% CI** | **P-value** |
| --- | --- | --- | --- | --- |
| **MCS child reported been bullied by age 7 (primary outcome)** |  |  |  |  |
| Highest | **Reference** |  |  |  |
| 4 | 1.05 | 0.98 | 1.13 | <0.001 |
| 3 | 1.13 | 1.05 | 1.21 |  |
| 2 | 1.13 | 1.05 | 1.22 |  |
| 1 | 1.21 | 1.12 | 1.31 |  |
| **PRIOR FACTORS *- to be adjusted for at baseline*** | | | | |
| Child's sex (parent reported)** |  |  |  |  |
| Male | 1.12 | 1.07 | 1.17 | <0.001 |
| Child: minority/ ethnic at birth (parent reported) |  |  |  |  |
| Other | 1.02 | 0.95 | 1.09 | 0.6284 |
| **FACTORS INFLUENCING SOCIAL NETWORK** | | | | |
| Child friendships at 5yrs old (parent reported) |  |  |  |  |
| Has no close friends | 1.17 | 0.97 | 1.41 | 0.0985 |
| Like to play with friends outside of school at 5yrs old (parent reported) |  |  |  |  |
| No | 0.91 | 0.85 | 0.97 | 0.005 |
| After school sport at 5yrs old (parent reported) |  |  |  |  |
| 3 or more days per week | **Reference** |  |  |  |
| 2 days per week | 0.89 | 0.81 | 0.97 | <0.001 |
| 1 day per week | 0.86 | 0.80 | 0.92 |  |
| Less often | 0.91 | 0.87 | 0.96 |  |
| Family's social network at 5yrs old (parent reported) |  |  |  |  |
| Both family and friends nearby | **Reference** |  |  |  |
| Family nearby | 1.10 | 1.02 | 1.19 | 0.0189 |
| Friends nearby | 1.02 | 0.96 | 1.08 |  |
| No family and friends nearby | 1.08 | 1.01 | 1.16 |  |
| Moved school ever at 5yrs old (parent reported) |  |  |  |  |
| Yes | 1.04 | 0.91 | 1.19 | 0.5581 |
| **FACTORS INFLUENCING EARLIER LIFE FAMILY RELATIONSHIPS** | | | | |
| Number of children in household at MCS birth (parent reported) |  |  |  |  |
| Only MCS child | **Reference** |  |  | 0.7225 |
| 2 children in household (inc. MCS child) | 0.95 | 0.91 | 0.99 |  |
| 3+ children in household (inc. MCS child) | 0.98 | 0.90 | 1.07 |  |
| Main responder's relationship at 3yrs old (parent response to Pianto scale) |  |  |  |  |
| Not warm | 1.16 | 0.95 | 1.41 | 0.1585 |
| Paternal relationship at 3yrs old (parent response to Pianto scale) |  |  |  |  |
| Not warm | 1.21 | 0.97 | 1.51 | 0.0853 |
| Parenting style at 3yrs old (parent reported) |  |  |  |  |
| Formal | **Reference** |  |  |  |
| Informal | 0.99 | 0.94 | 1.04 | 0.6253 |
| Family break-up from 3 or 5yrs old (parent reported) |  |  |  |  |
| Yes | 1.20 | 1.15 | 1.25 | <0.001 |
| Main responder's levels of distress at 5yrs old (parent response to Kessler scale) |  |  |  |  |
| Distressed (Kessler score >5) | 1.14 | 1.08 | 1.21 | <0.001 |
|  |  |  |  |  |
| Distressed (Kessler score >5) | 1.06 | 0.99 | 1.14 | 0.0816 |
| Smacking used as discipline at 5yrs old (parent reported) |  |  |  |  |
| Never | **Reference** |  |  | 0.0209 |
| Less than monthly | 1.06 | 1.01 | 1.11 |  |
| More than monthly/ daily | 1.11 | 0.94 | 1.31 |  |
| Frequency family indoor activities at 5yrs old (parent reported) |  |  |  |  |
| Daily/several times per week | **Reference** |  |  |  |
| Monthly/every few months | 1.01 | 0.97 | 1.06 | 0.001 |
| Annually/ never | 1.32 | 1.14 | 1.53 |  |
| **FACTORS FOR EARLIER LIFE ABILITIES AND BEHAVIOURS** | | | | |
| School readiness |  |  |  |  |
| No | 1.16 | 1.09 | 1.24 | <0.001 |
| Total Strength and Difficulty Score at age 3 years old |  |  |  |  |
| Average | **Reference** |  |  | <0.001 |
| Borderline | 1.27 | 1.17 | 1.38 |  |
| Below average | 1.31 | 1.21 | 1.42 |  |
| Child has a limiting long term condition at age 5 years old |  |  |  |  |
| Yes | 1.04 | 0.99 | 1.10 | 0.1334 |
| Body Mass Index at age 5 years old |  |  |  |  |
| Normal | **Reference** |  |  | 0.0174 |
| Overweight | 1.07 | 1.00 | 1.13 |  |
| Obese | 1.08 | 1.02 | 1.15 |  |
