# Supplementary material for: Understanding social inequalities in children being bullied: UK Millennium Cohort Study findings
Source: PLoS One. 2019 May 29;14(5):e0217162. doi: 10.1371/journal.pone.0217162 (PMC6541267; doi:10.1371/journal.pone.0217162)
Supplement: S3 Table — presents the risk ratios (RR) 95% confidence intervals for multiple imputations by chained equations, alternative outcome and alternative exposures. (DOC) [file pone.0217162.s004.doc]

**S4 Table.** Results from the formal counterfactual medication analysis.

| **INDEPENDENT MODELS** | **Total effect**  **RR** | **95% LCI** | **95% UCI** |
| --- | --- | --- | --- |
| Baseline (adj. sex/ ethnicity) | 1.20 | 1.06 | 1.38 |
| Social network (Model 1) | 1.19 | 1.05 | 1.35 |
| Family relations (Model 2) | 1.16 | 1.02 | 1.32 |
| Child characteristics and abilities (Model 3) | 1.13 | 0.99 | 1.30 |
| Final model* | 1.11 | 0.97 | 1.28 |

**all risk factors from social network, family relationships and child abilities and behaviours domains*

S4 show the findings from applying a formal counterfactual medication analysis (Medflex). This approach gives us the flexibility to assess the effect of specific causal pathways in order to quantify its contribution to the outcome of interest.
