# Supplementary material for: Understanding social inequalities in children being bullied: UK Millennium Cohort Study findings
Source: PLoS One. 2019 May 29;14(5):e0217162. doi: 10.1371/journal.pone.0217162 (PMC6541267; doi:10.1371/journal.pone.0217162)
Supplement: S4 Table — show the findings from applying a formal counterfactual medication analysis, an approach that gives us the flexibility to assess the effect of specific causal pathways in order to quantify its contribution to the outcome of interest. (DOC) [file pone.0217162.s005.doc]

S3 Table Robustness test for being bullied at age 7 and socio-economic circumstances (SEC) in the multivariable analysis stage: presenting risk ratios (RR) 95% confidence intervals for multiple imputations by chained equations, alternative outcome and an alternative exposures.

|  | **ADJUSTED**  **BASELINE*** | | | **MODEL 1**  **SOCIAL NETWORK** | | | **MODEL 2**  **FAMILY RELATIONS** | | | **MODEL 3**  **CHILD ABILITIES**  **& BEHAVIOURS** | | | | **FINAL MODEL**** | | |
| --- | --- | --- | --- | --- | --- | --- | --- | --- | --- | --- | --- | --- | --- | --- | --- | --- |
|  | **RR** | **95% LCI** | **95%**  **UCI** | **RR** | **95% LCI** | **95% UCI** | **RR** | **95% LCI** | **95%**  **UCI** | **RR** | | **95%**  **LCI** | **95%** | **RR** | **95% LCI** | **95% UCI** |
|  |  |  |  |  |  |  |  |  |  |  |  |  | **UCI** |  |  |  |
| Multiple Imputed dataset for household income and being bullied at age 7 (N=10,560) | | | | | | | | | | | | | | | | |
| P-value |  | **<0.001** |  |  | <0.001 |  |  | <0.001 |  | |  | **<0.001** |  |  | **0.004** |  |
| Highest income quintile | Ref | - | - | Ref | - | - | Ref | - | - | | Ref | - | - | Ref | - | - |
| 4 | 1.06 | 0.97 | 1.16 | 1.05 | 0.96 | 1.15 | 1.06 | 0.97 | 1.16 | | 1.06 | 0.97 | 1.15 | 1.05 | 0.96 | 1.15 |
| 3 | 1.12 | 1.03 | 1.22 | 1.1 | 1.01 | 1.21 | 1.11 | 1.02 | 1.22 | | 1.11 | 1.02 | 1.21 | 1.1 | 1.00 | 1.2 |
| 2 | 1.13 | 1.03 | 1.23 | 1.1 | 1.00 | 1.2 | 1.12 | 1.03 | 1.23 | | 1.09 | 1.00 | 1.19 | 1.08 | 0.98 | 1.19 |
| Lowest income quintile | 1.23 | 1.13 | 1.34 | 1.18 | 1.08 | 1.3 | 1.21 | 1.11 | 1.33 | | 1.18 | 1.07 | 1.29 | 1.15 | 1.04 | 1.27 |
| Persistent bullying as an alternative outcome and household income (N=5,857) | | | | | | | | | | | | | | | |  |
| **P-value** |  | **<0.001** |  |  | **<0.001** |  |  | **<0.001** |  | |  | **<0.001** |  |  | 0.003 |  |
| Highest income quintile | Ref | - | - | Ref | - | - | Ref | - | - | | Ref | - | - | Ref | - | - |
| 4 | 1.36 | 0.96 | 1.93 | 1.31 | 0.93 | 1.84 | 1.29 | 0.91 | 1.84 | | 1.32 | 0.93 | 1.87 | 1.23 | 0.87 | 1.74 |
| 3 | 1.99 | 1.44 | 2.73 | 1.82 | 1.33 | 2.48 | 1.85 | 1.35 | 2.54 | | 1.81 | 1.31 | 2.51 | 1.61 | 1.18 | 2.2 |
| 2 | 2.84 | 2.05 | 3.95 | 2.5 | 1.75 | 3.57 | 2.48 | 1.77 | 3.47 | | 2.41 | 1.72 | 3.38 | 1.99 | 1.37 | 2.89 |
| Lowest income quintile | 3.07 | 2.11 | 4.47 | 2.64 | 1.76 | 3.96 | 2.71 | 1.83 | 4.01 | | 2.41 | 1.63 | 3.55 | 1.97 | 1.27 | 3.05 |
| Maternal Education at MCS child's birth as an alternative SEC exposure and for being bullied at age 7 (N= 5,772) | | | | | | | | | | | | | | |  |  |
| P-value |  | 0.068 |  |  | 0.192 |  |  | 0.291 |  | |  | 0.412 |  |  | 0.805 |  |
| Degree plus | Ref | - | - | Ref | - | - | Ref | - | - | | Ref | - | - | Ref | - | - |
| Diploma | 1.05 | 0.93 | 1.18 | 1.06 | 0.94 | 1.19 | 1.04 | 0.93 | 1.17 | | 1.04 | 0.92 | 1.16 | 1.04 | 0.93 | 1.17 |
| A-Levels | 1.06 | 0.97 | 1.16 | 1.05 | 0.92 | 1.18 | 1.03 | 0.91 | 1.17 | | 1.03 | 0.91 | 1.16 | 1.02 | 0.91 | 1.16 |
| GCSE A-C | 1.12 | 1.01 | 1.25 | 1.05 | 0.96 | 1.15 | 1.03 | 0.94 | 1.13 | | 1.03 | 0.94 | 1.13 | 1.02 | 0.93 | 1.11 |
| GCSE D-G | 1.13 | 0.99 | 1.28 | 1.11 | 1.00 | 1.23 | 1.09 | 0.98 | 1.21 | | 1.07 | 0.96 | 1.19 | 1.04 | 0.94 | 1.17 |
| None | 1.18 | 1.12 | 1.25 | 1.1 | 0.96 | 1.26 | 1.08 | 0.94 | 1.23 | | 1.06 | 0.93 | 1.2 | 1.02 | 0.88 | 1.17 |
| Without partner reported outcomes: SEC exposure and for being bullied at age 7 (N= 7887) | | | | | | | | | | | | | | |  |  |
| **P-value** | **<0.001** | | | **<0.001** | | | **<0.001** | | | | **<0.001** | | | **0.002** | | |
| Highest income quintile | Ref | - | - | Ref | - | - | Ref | - | - | | Ref | - | - | Ref | - | - |
| 4 | 1.07 | 0.99 | 1.17 | 1.07 | 0.98 | 1.16 | 1.07 | 0.98 | 1.16 | | 1.06 | 0.98 | 1.15 | 1.06 | 0.98 | 1.15 |
| 3 | 1.15 | 1.06 | 1.25 | 1.14 | 1.05 | 1.24 | 1.14 | 1.04 | 1.24 | | 1.13 | 1.04 | 1.23 | 1.12 | 1.03 | 1.23 |
| 2 | 1.15 | 1.05 | 1.25 | 1.12 | 1.02 | 1.23 | 1.13 | 1.03 | 1.23 | | 1.10 | 1.01 | 1.21 | 1.08 | 0.99 | 1.19 |
| Lowest income quintile | 1.25 | 1.14 | 1.37 | 1.22 | 1.11 | 1.34 | 1.22 | 1.11 | 1.34 | | 1.20 | 1.09 | 1.31 | 1.17 | 1.06 | 1.29 |

*sex and ethnicity adjusted ** All three domains combined: social network, family relationships and the MCS child’s abilities and behaviours
